# Diagnosis and characterization of canine distemper virus through sequencing by MinION nanopore technology

**DOI:** 10.1038/s41598-018-37497-4

**Published:** 2019-02-08

**Authors:** Alessia Peserico, Maurilia Marcacci, Daniela Malatesta, Marco Di Domenico, Annamaria Pratelli, Iolanda Mangone, Nicola D’Alterio, Federica Pizzurro, Francesco Cirone, Guendalina Zaccaria, Cesare Cammà, Alessio Lorusso

**Affiliations:** 1 National Reference Center for Whole Genome Sequencing of microbial pathogens: database and bioinformatic analysis, Istituto Zooprofilattico Sperimentale dell’Abruzzo e del Molise, Campo Boario, 64100 Teramo, Italy; 2 Dipartimento di Medicina Veterinaria, strada provinciale per Casamassima Km 3, 70100 Valenzano, Bari Italy

## Abstract

Prompt identification of the causative pathogen of an infectious disease is essential for the choice of treatment or preventive measures. In this perspective, nucleic acids purified from the brain tissue of a dog succumbed after severe neurological signs were processed with the MinION (Oxford Nanopore Technologies, Oxford UK) sequencing technology. Canine distemper virus (CDV) sequence reads were detected. Subsequently, a specific molecular test and immunohistochemistry were used to confirm the presence of CDV RNA and antigen, respectively, in tissues. This study supports the use of the NGS in veterinary clinical practice with potential advantages in terms of rapidity and broad-range of molecular diagnosis.

## Introduction

Rapid identification of the etiologic agent of an infectious disease is essential for setting up treatment and preventive measures. In general, pathogen identification is performed by direct diagnostic tests which normally include amplification of target nucleic acids by PCR-based assays. Although these approaches are highly specific and often validated, they suffer a number of limitations, including the difficulties of testing for the plethora of rare pathogens that might be expected to cause a given pathology and their inability to identify new or unexpected pathogens, eventually originating from cross-species jumps. Therefore, the availability of other more rapid, broad-range techniques has become more and more important in the milieu of laboratory diagnosis of infectious diseases.

In this context one of these novel and intriguing technologies is certainly represented by next generation sequencing (NGS), even more when performed for direct identification of pathogens from clinical samples. Genome sequencing directly from biological samples can provide insights into how viruses transmit and spread, and it has been successfully applied to both virus discovery and diagnostics^1–3^. Recently the release of the MinION (Oxford Nanopore Technologies, Oxford, UK), a novel portable real-time NGS sequencer, enables the application of sequencing for rapid diagnosis with inexpensive sample preparation even in low-throughput laboratories^4–10^.

Canine distemper (CD) is a highly contagious disease which involves mainly young dogs and other susceptible carnivores with high morbidity and mortality rates. CD infection is characterized by lympho-, neuro- and epithelio-tropism resulting in systemic infection with severe clinical signs and death^11,12^.

The causative agent is canine distemper virus (CDV), a member of the genus *Morbillivirus* of the family *Paramyxoviridae*. CDV is an enveloped, negative-sense, single-stranded RNA virus. Like other paramyxoviruses, the virus contains six structural proteins, termed nucleocapsid (N), phospho (P), large (L), matrix (M), hemagglutinin (H) and fusion (F) protein, and two accessory non-structural proteins (C and V) that were found as extra-transcriptional units within the P gene^13^. Circulating CDV strains are grouped in geographical lineages based on the genetic correlation with the H protein encoding gene. Phylogenetic and molecular evolutionary analyses of CDV have revealed that mutations affecting the binding site of the H protein for virus entry receptors are associated with the occurrence of disease emergence in novel host species^14^. In Italy, CDV has been detected in several occasions with different viral strains belonging to separate lineages circulating in the field^15–22^. Generally, in Italy CDV circulation in domestic dogs is under control as for the availability of valid vaccines on the market. However, occasional outbreaks occur, and these outbreaks are generally related with the illegal trade of infected dogs from Eastern Europe^17,18,21,23^. The last large CDV outbreak in Italy was caused by a strain belonging to the Arctic lineage (prototype CDV2784/2013). It occurred in 2013 involving primarily the Abruzzi region, an area hosting several natural parks with significant animal biodiversity. In this outbreak, distemper caused overt disease in unvaccinated domestic and shepherd dogs, Apennine wolves (*Canis lupus*) and other wild carnivores living in natural areas of the Abruzzi and neighboring regions, including Molise. CDV circulation was still evidenced in the following years in wild and domestic animals^22^. Direct diagnosis of CDV infection in animals showing neurological signs is primarily performed by molecular assays detecting the viral genome in the cerebrospinal fluid or alternatively in the urine^20,24^.

In this manuscript, we described the identification and characterization of a CDV strain from the brain tissue of a dog by using the MinION sequencing Nanopore technology.

## Results

### MinION real time sequencing from the brain tissue

**Figure 1:**
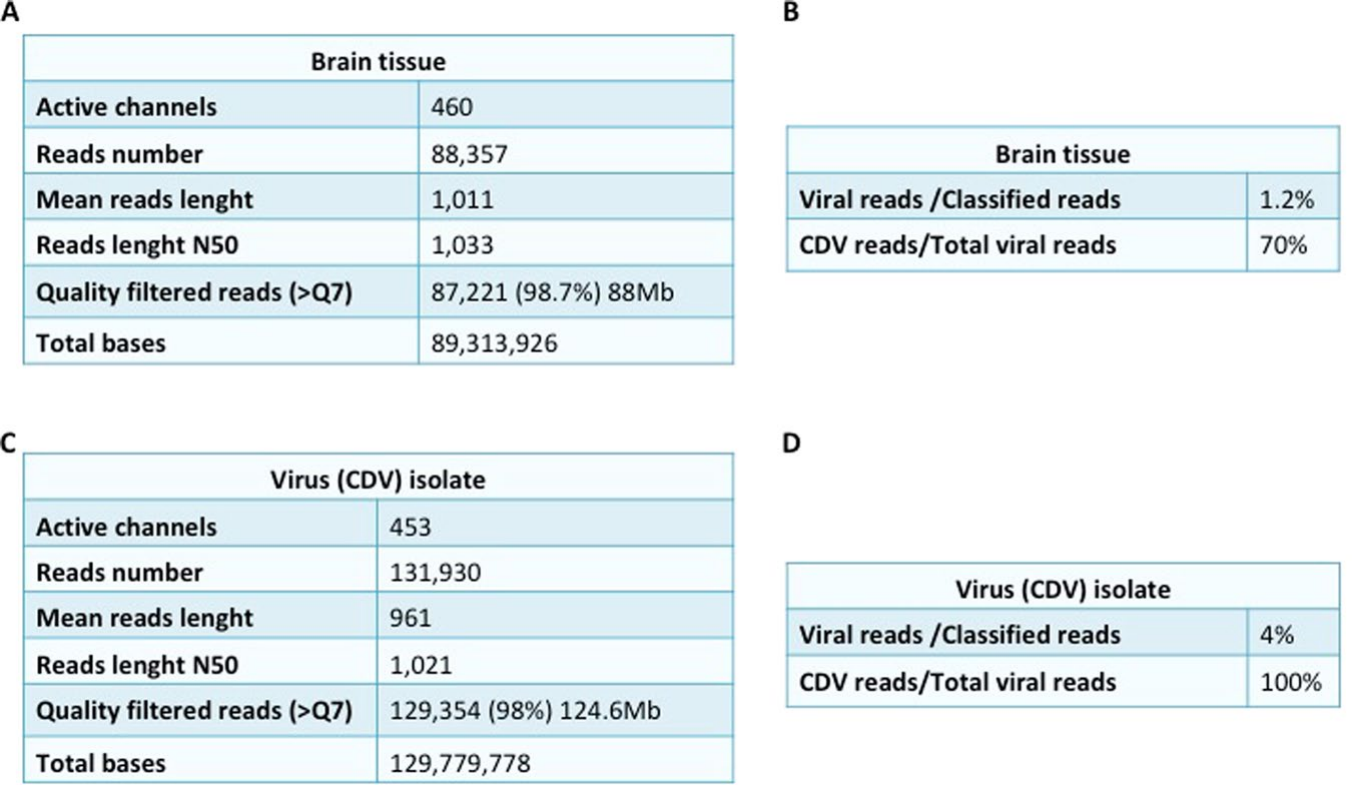
Statistics of Nanopore sequencing data from the brain tissue (**A**,**B**) and the CDV isolate (**C**,**D**). These data were obtained by using NanoPlot.

**Figure 2:**
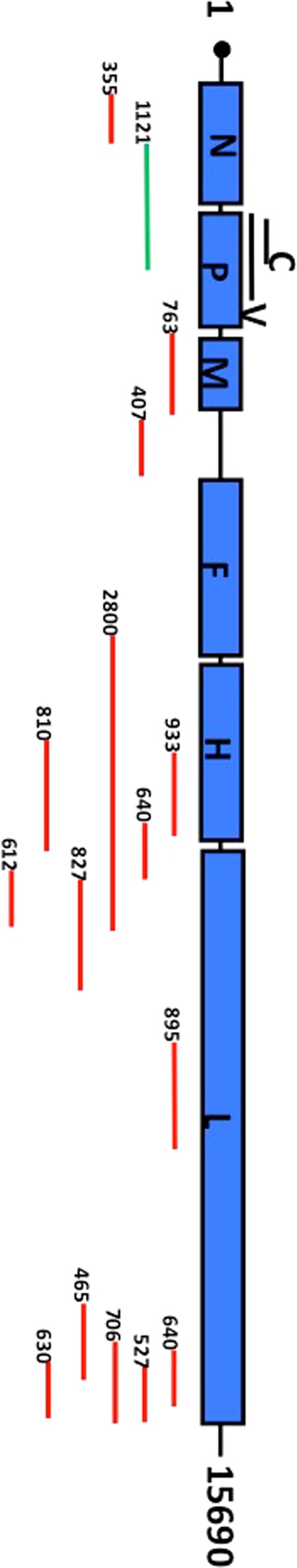
Graphic representation of CDV genome (15.690 bp) and mapping of raw sequence reads originated from the MinION run with nucleic acid purified from the brain tissue. The green bar indicates the first detected CDV raw read. CDV gene sequences coding for viral proteins are represented by blue boxes. The number next to each bar/read indicates the nt length.

Data were produced by 460 active channels of the flow cell. A total number of 88.357 reads (89.313.926 bp) were obtained from the cDNA sequencing run. By assessing a quality cut-off (>Q7), a total number of 87.221 reads of sequencing reads was selected. The average length of the reads was 1.011 nt. Of these 1.886, the 96,4% was classified as eukaryota, the 2,4% as bacteria and the 1,2% as viruses (Fig. 1A,B). A total number of 16 reads, representing the 70% of reads assigned to viruses, were further classified as canine distemper virus. These reads contained sequence information of all protein encoding genes of CDV genome, in particular, H and L sequences (Fig. 2). One read of 2.688 nt encompassed a portion of the F, the whole H and of a portion of the L coding sequence. The first of these reads, which encompassed the N and P gene sequences, (Fig. 2) was retrospectively shown to be acquired within the first 20 minutes of sequencing run, the last after 11 hours. Overall, the 50% of CDV reads was revealed within the first 5 hours of real time sequencing. Conversely, within the run performed with the DNA library, no reads were classified as viruses (data not shown).

### rtPCR_CDV_, IHC and isolation

**Figure 3:**
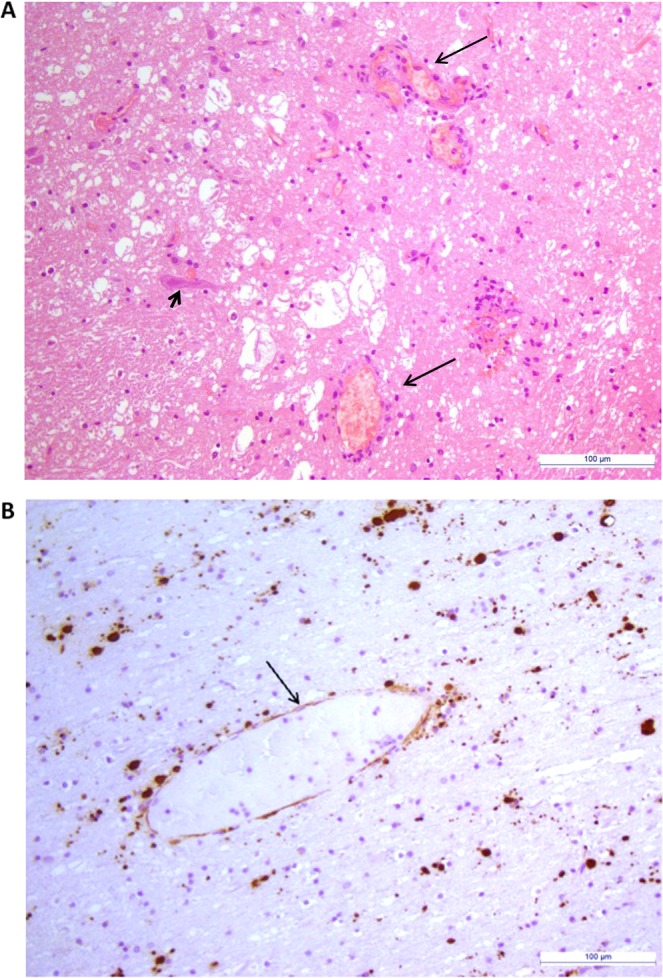
(**A**) Brain, acute demyelinating encephalitis with vacuolation of the white matter due to myelin sheath oedema. Acidophilic viral intranuclear inclusion in a shrunken neuron (black arrow head). Perivascular inflammatory changes are minimal with few cells (black arrows). HE staining. Bar = 100 µm. (**B**) Brain, strong staining of CDV antigen in neurons and in endothelial cells of a blood vessel (black arrow). IHC. Bar = 100 µm.

A specific molecular test was performed after the identification of CDV by MinION. The rtPCR_CDV_ confirmed the presence of RNA belonging to CDV in both tissues. Threshold cycles (C_*T*_) were 24 and 22 from brain and lung, respectively. Histological lesions and visualization of CDV antigen are showed in Fig. 3A,B, respectively. CDV (strain CDV1747/ITL2018) was isolated only from the lung homogenate at the second cell passage onto Vero.DogSLAMtag-cells and confirmed by rtPCR_CDV_.

### Whole genome sequencing of the isolate by MinION

**Figure 4:**
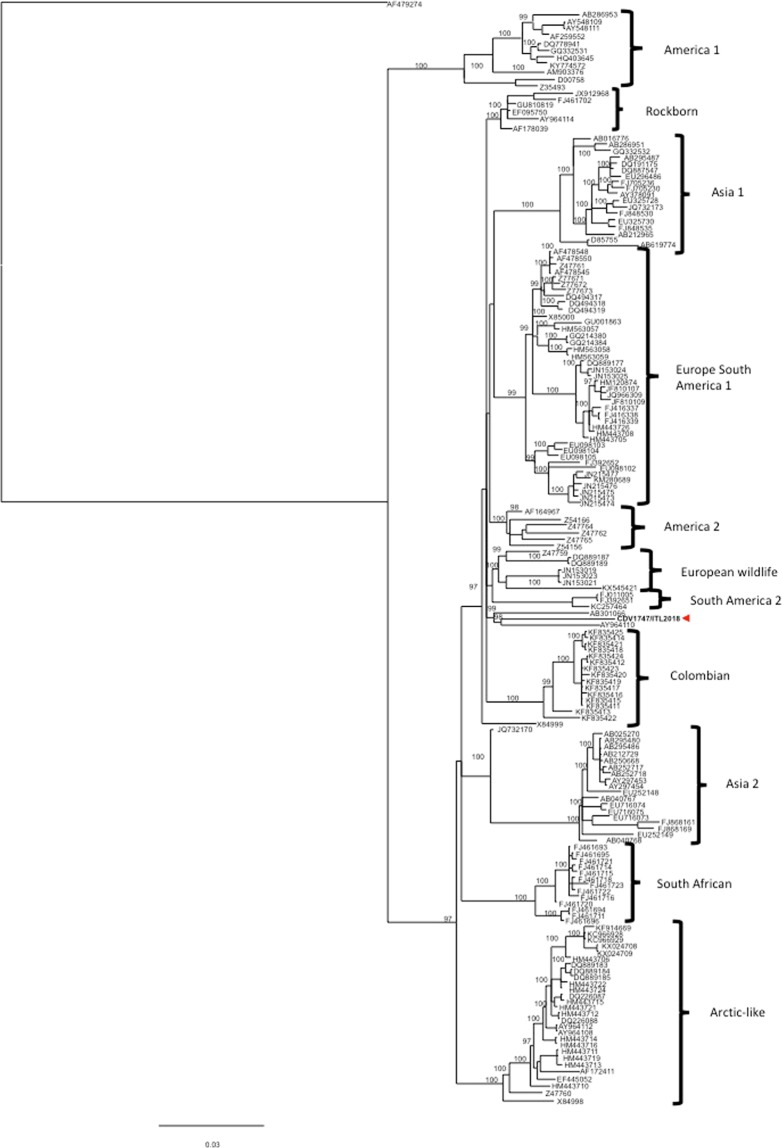
Phylogenetic analysis of H protein encoding gene sequences retrieved from GenBank. Accession numbers are provided. Philogeny carried out by Mr Bayes program implemented within the software package Geneious [version 9.1.8]. Bayesian inference was performed using four “chains” run over one million generations (with the first 2000 trees discarded as ‘burn-in) and supplying statistical support with subsampling over 200 replicates. jModelTest was used to identify the most appropriate model of evolution. The identified program settings for all partitions, under the Akaike Information Criteria, included six-character states (General Time Reversible model), a proportion of invariable sites, and a gamma distribution of rate variation across sites (GTR + I + G). H gene sequence of Phocine distemper virus has been used as outgroup. Strain CDV1747/ITL2018 is indicated by a red arrow. Putative CDV lineages are indicated by brackets. Bar indicates the estimated number of nt substitutions per site.

The whole genome sequence of CDV1747/ITL2018 (cell passage 3 onto Vero.DogSLAMtag-cells) has been obtained by an additional MinION sequencing run. Data were produced using 453 active channels of the flow cell. A total number of 131.930 reads were obtained from the cDNA sequencing run. By assessing a quality cut-off (>Q7), a total number of 129.354 reads (129.779.778 bp) of sequencing reads was selected. The average of the length of the reads was 961 nt. A total number of 5.164 reads, representing the 100% of the virus-assigned reads, were classified as canine distemper virus (Fig. 1C,D). Genomic coverage was 295X with 30X of minimum depth. The consensus sequence of CDV1747/ITL2018 (15.690 nt in length) has been deposited and annotated with the GenBank database (acc. no. MH316137). Raw reads, obtained from the MinION run with nucleic acids purified from the brain tissue, showed a nt identity ranging from 75 to 93% with the deposited consensus sequence. On the other hand, nt identity between the H protein encoding gene sequence of the isolate obtained by MinION and the sequences (either from isolate or infected tissues) obtained by Sanger sequencing was 100%. According to the phylogenetic analysis (Fig. 4) of the H protein encoding gene, CDV1747/ITL2018 clusters with strains Th270L and 19.876 identified from dogs in Thailand and USA, respectively. The H protein encoding gene sequence of CDV1747/ITL2018 bears, indeed, the highest sequence identity with strains Th270L (96.29%) and 19876 (96.59%) whereas with reference CDV lineages and vaccine strains, including CDV599/2016 (Europe wildlife lineage)^22^ and CDV2784/2013 (Arctic lineage)^21,25^ which have been recently identified in central and southern Italy, sequence identity ranges from 90.89% to 95.48% (Table 1). Considering also the close temporal relationship between the onset of clinical signs and arrival to Italy, it is tempting to speculate that CDV1747/2018 was likely introduced to Italy from Cuba.

**Table 1:** Nucleotide (nt) sequence identity (expressed as %) between the H protein gene sequence of CDV1747/ITL2018 and representative strains belonging to existing CDV lineages.

CDV strains	CDV1747/2018	Th270L	19876	Snyder Hill	Rockborn	Yanaka	SNP350/09/ITA	A75/17	CDV599/2016	Argentina 26	39-CO/12	07D111	4L70214	CDV2784/2013
	nt	nt	nt	nt	nt	nt	nt	nt	nt	nt	nt	nt	nt	nt
CDV1747/ITL2018	/	96.29	96.59	90.89	95.48	93.11	93.72	95.29	93.05	93.47	93.23	92.08	93.05	92.44
Th270L	96.29	/	96.09	91.01	95.42	94.23	94.11	95.49	93.81	94.29	94.66	92.35	93.69	93.02
19876	96.59	96.09	/	91.07	95.32	93.93	93.81	95.81	93.69	94.49	94.29	92.71	93.38	92.71
Snyder Hill	90.89	91.01	91.07	/	93.14	91.20	91.38	92.59	90.95	91.74	91.86	90.89	92.05	91.20
Rockborn	95.48	95.42	95.32	93.14	/	96.54	96.84	98.48	96.05	96.66	96.66	94.84	96.11	95.08
Yanaka	93.11	94.23	93.93	91.20	96.54	/	94.54	96.17	94.11	94.35	94.47	93.08	94.29	92.96
SNP350/09/ITA	93.72	94.11	93.81	91.38	96.84	94.54	/	96.24	94.29	94.54	94.74	92.90	94.11	93.26
A75/17	95.29	95.49	95.81	92.59	98.48	96.17	96.24	/	95.63	96.24	96.30	94.35	95.69	94.66
CDV599/2016	93.05	93.81	93.69	90.95	96.05	94.11	94.29	95.63	/	94.17	94.11	92.29	93.62	92.77
Argentina 26	93.47	94.29	94.49	91.74	96.66	94.35	94.54	96.24	94.17	/	94.60	92.65	94.29	92.96
39-CO/12	93.23	94.66	94.29	91.86	96.86	94.47	94.74	96.30	94.11	94.60	/	93.02	94.60	93.14
07D111	92.08	92.35	92.71	90.89	94.84	93.08	92.90	94.35	92.29	92.65	93.02	/	93.62	92.65
4L70214	93.05	93.69	93.38	92.05	96.11	94.29	94.11	95.69	93.62	94.29	94.60	93.62	/	94.29
CDV2784/2013	92.44	93.02	92.71	91.20	95.08	92.96	93.26	94.66	92.77	92.96	93.14	92.65	94.29	/

## Discussion

This study was planned and performed to assess the feasibility of the MinION sequencing technology in routine diagnostics. In this occasion, CDV infection was identified in the brain tissue of a dog imported to Italy from Cuba. The dog died after severe neurological infection, which was evidenced two days after the arrival from Cuba.

The MinION, performed with nucleic acids purified from the infected brain tissue, was able to provide indication of CDV infection. A specific molecular assay and IHC were then performed to confirm the presence of CDV in tissues. The same NGS platform was also used to obtain the whole genome sequence of the isolate. The involved strain, CDV1747/ITL2018, has been shown to be different from CDV strains which have been demonstrated to circulate in central/southern Italy in recent years.

One could reasonably argue that the clinical signs were already satisfactory to address the molecular diagnosis of CDV by existing direct assays, including rtPCR_CDV_. Although this aspect is undeniably true, the intention of this study is to promote efforts to validate NGS into routine diagnostics in order to get as soon as possible the identification of the etiological agent of a given disease and potentially opportunistic pathogens as previously demonstrated to occur in the field^26^ with a broad-range diagnostic method. Undeniably, our study has some drawbacks. One of them may be represented by the low number of CDV reads originating from the MinION run which may apparently underestimates its use into diagnostic according to the adopted protocol. This could be explained by the fact that we did not perform any viral enrichment. In this regard, novel techniques for viral enrichment from stools, environmental and tissue samples have been recently described for viral metagenomics studies with second-generation sequencing technologies^27–29^. As for today, further efforts are still necessary to assess protocols for virome preparation for third-generation sequencing platforms. Indeed, although Theuns and colleagues^7^ described a viral enrichment protocol from stool samples for MinION sequencing^5^, a specific viral enrichment from tissue samples combined with MinION still needs to be explored.

On the other hand, in this study we performed the SISPA amplification before the sequencing run. SISPA is an established protocol which permitted, through random primer amplification, the identification and characterization of several viruses, including different avian RNA viruses^30^, Schmallenberg virus^31^, Hepatitis C virus from clinical biopsy samples^32^, a novel mink astrovirus^33^, viruses from human stool samples^34^, porcine epidemic diarrhea virus from swine feces^35^, classical and atypical Bluetongue virus serotypes from blood samples^36–39^, feline morbillivirus from urine^40^ and viruses responsible for respiratory tract infections^41^. Moreover, recently our group combined successfully SISPA and MinION sequencing for the identification of bovine enterovirus from a cell-culture isolate^6^. By the way, several enrichment strategies from tissues and combination of MinION sequencing are currently under investigation by our team. Furthermore, we did not perform live basecalling and diagnostic indications were provided after the sequencing run was stopped without live acquisition of raw sequence data. The local WIMP analysis, indeed, was completed after 13 hours starting from the load of the flow cell with cDNA. However, the retrospective analysis showed that the first read came out within the first 20 minutes of the sequencing run. Interestingly, when the sample was re-processed by the live pipeline (mimicking a real-time sequencing scenario) the first sequence read was assigned to CDV after 40 minutes from the beginning of the sequencing run. This would suggest that if used as point-of-care device, solid identification of CDV would have been acquired within less than 1 hour starting from the beginning of the sequencing run.

Conversely, what could have happened if rtPCR_CDV_ was negative when used as first diagnostic attempt? The only valid strategy for diagnosis would have been the one which includes the testing of purified nucleic acids for all the plethora of pathogens known to cause the clinical signs observed in that dog. Reasonably, this approach would have required a longer period to get, eventually, a valid response. This aspect combined with the high chance of unavailability of specific tests, contributes to the relatively high proportion of cases that remain undiagnosed. Therefore, we attempted to use the MinION sequencing platform to get diagnostic indications. In this case, acquired raw CDV reads were not identical to the deposited consensus sequence. This can be easily explained by the fact that reads originated by the second MinION run, with nucleic acids purified from the viral isolate, were further processed with two bioinformatics, GraphMap and Pilon, tools. These tools fix the errors which occurred during the assembly of sequence reads. Further support to the credibility of the final consensus sequence has been provided by the Sanger sequencing of the H protein encoding gene of the occurring strain, either from the isolate or directly from the tissue specimens. Indeed, Sanger sequencing evidenced 100% of nt identity with the sequence obtained by the second MinION run. Nevertheless, raw reads were indispensable and satisfactory to identify the involved viral species and to get diagnostic indications.

The MinION platform is a unique, highly portable, single molecule real time sequencer currently available. This technology recently emerged to reduce the sequencing costs and to simplify the library preparation procedures. The hallmark of the MinION technology is certainly represented by the fact that this platform can be routinely used to investigate viral and bacterial outbreaks also in low throughput laboratories and, importantly, in field conditions with a real time acquisition of raw data. Further validation experiments are currently ongoing to validate the use of MinION in the daily routine with different biological matrices. Overall, these data demonstrate that MinION is affordable and able to detect CDV quickly and with enough accuracy, supporting its potential for virus surveillance also in field conditions.

## Methods

### Case description

A male of Mexican hairless dog (Xoloitzcuintli) born on July 4^th^, 2017 was legally introduced from Cuba to Italy (municipality of Campobasso, Molise region, southern Italy) on February 19^th^, 2018. The dog was vaccinated for rabies (Virbac-UK, https://uk.virbac.com/home/products/vaccines/main/dog-vaccines/canigen-rabies.html) on November 10^th^, 2017. On February 21^st^, 2018, two days after the arrival in Italy, the dog showed labored breathing and severe neurological signs including disorientation, lack of alertness, convulsive movements of the head and paws (Supplementary Video S1), aimless wandering, hyper-salivation and chewing movements of the jaw. Therapy consisted of administration of fluids, cortisone and antibiotics. However, the dog succumbed few days later. The carcass was sent to IZSAM for necropsy. A complete necropsy examination was accomplished at IZSAM. Lung and brain tissues were employed for diagnosis.

### **Virus isolation**

Vero cells stably expressing canine SLAM (Vero. DogSLAMtag^42^) were inoculated with lung and brain homogenates and incubated at 37  °C with 5% CO_2_ for a maximum of 5–7 days until cytopathic effect (CPE) was observed. Plates showing no CPE were further passaged up to passage 3.

### **Shotgun metagenomics by MinION**

30 mg of brain were chopped into small pieces with a razor blade, resuspended in 1 ml of Phosphate-buffered saline (PBS) and homogenized with a TissueLyser II (Qiagen, Hilden Germany) for 5 min following manufacturer’s guidelines.

The homogenate was centrifuged and 200 μl of the supernatant was used for nucleic acid purification using High Pure Viral Nucleic Acid Kit (Roche, Basel Swiss) and metagenomic analysis by MinION (Oxford Nanopore Technologies, Oxford UK). Nucleic acid elution was divided in two aliquots to perform RNA and DNA sequencing in two different runs as described in a previous study from our group with some modifications^6^.

Likewise, the supernatant of the Vero.DogSLAMtag-cells CDV isolate (passage 3) was collected and processed for RNA purification (High Pure Viral Nucleic Acid Kit, Roche, Basel CH).

RNA samples were processed by means of the Sequence Independent Single Primer Amplification (SISPA) method^43^ with some modifications to obtain cDNA^39,40^. FR26RV-N (GCCGGAGCTCTGCAGATATCNNNNNN) and FR20RV (GCCGGAGCTCTGCAGATATC) primers were used for SISPA protocol^43^. Reverse transcriptase and PCR were carried out by using the SuperScript II Reverse Transcriptase (Thermo Fisher Scientific) and Pfu Ultra II Fusion HS DNA Polymerase (Agilent), respectively.

DNA and amplified cDNA were quantified by Qubit dsDNA HS assay (Thermo Fisher Scientific, Waltham, MA) and used for library preparation by Low input genomic DNA by PCR Barcoding (SQK-LWB001, Oxford Nanopore Technologies, Oxford UK) following manufacturer’s guidelines. Briefly, DNA ends were end-repaired and dA-tailed using the NEBNext End Repair/dA tailing module (New England Biolabs, Hitchin, UK). PCR adapters were ligated onto the prepared ends using the Blunt/TA Ligase Master Mix (New England Biolabs, Hitchin, UK) and a unique barcode was added by PCR. The amplification program consisted of a first 3 min step at 95 °C followed by 14 cycles with the following conditions: 95 °C for 15 sec, 56 °C for 15 sec and 65 °C for 6 min. The program ended with 1 step at 65 °C for 6 min. Sequencing adapters were added prior library loading on the Flow cell MIN106, R9 version (Oxford Nanopore Technologies). All purification steps were carried out using AMPure XP beads (Agencourt, Beckman Coulter Brea, CA) according the SQK-LWB001 sequencing protocol. For sequencing, the NC_48hr_sequencing_FLO-MIN106_SQK-LBW001 program was run on MinKNOW Software v.1.4.2. The sequencing run was stopped after 11 hours form starting sequencing.

### **MinION data analysis**

Fast5 raw MinION files were processed locally by using the Albacore software (version 2.1.10) to obtain fastq files output with a quality cut-off (>Q7). The local base calling allowed to obtain fastq files within less than 2 hours of processing. “What is in my pot” (WIMP) workflow^43^ was then initiated in the EPI2MEAgent Software (version 2.47.537208) for analyzing brain tissue originated-reads produced by Albacore. Briefly, WIMP tool enables the comparison in real time of the raw reads against the NCBI Organism Database; this tool classifies and identifies the species by looking at the k-mers produced. WIMP platform required 10 minutes of analysis for classification.

Reads derived from the CDV isolate sequencing run, after Albacore processing, were further demultiplexed, and trimmed by using Porechop (version 0.2.3). To obtain a draft sequence by mapping, the closest reference sequence was identified performing Blastn search (version 0.5.2). The consensus sequence was obtained using the GraphMap (version 0.4.1^44^) aligner and using Pilon software (version 1.22). Specifically, we used the default parameters for Graphmap and a combination of samtools, bcftools, vcfutils and internal scripts to extract the consensus sequence from the alignment. Then, consensus sequence was refined by Pilon. The final sequence and ORFs were also manually inspected using the Ugene software (version 1.29.0^45^). Quality check analysis of raw reads was performed by using the Nanoplot (version 1.17.2).

### Diagnostic RT-qPCR (rtPCR_CDV_), **immunohistochemistry(IHC)**

Purified nucleic acids from brain and lung were screened by rtPCR_CDV_ with specific primers and probe for the N protein-encoding gene of CDV^46^. The brain sample was fixed in 10% neutral buffered formalin and embedded in paraffin wax. Sections (5 μm) were stained with haematoxylin and eosin (HE). Additional tissue sections were also subjected to immunohistochemistry (IHC) using primary antibody specific for CDV (1:1000, mouse monoclonal antibody (Ab) anti CDVNP; VMRD™, Pulmann WA). Labeling was performed by using a biotinylated goat anti-mouse Ab and subsequently by indirect streptavidin-biotin method (Dako REAL™ Detection System, Peroxidase/DAB kit, Santa Clara CA, USA). Antigen retrieval was performed by heat treatment in citrate buffer pH 6.0, 0.01 M at 121 °C for 5 min. Sections were visualized with 3,3 diaminobenzidine solution (DAB, Dako Santa Clara CA, USA), and finally counterstained with Mayer’s hematoxylin. CDV-positive lung tissue sections of dogs available *in house* were used as positive controls, whereas the specificity of the immune-labeling was verified with an unrelated Ab.

### **CDV isolate and phylogeny**

RNAs purified from the CDV isolate (passage 3) and tissue specimens were used for RT-PCR and Sanger sequencing of the H protein encoding sequence. Amplicons were obtained using RH-3 and RH-4 primers^47^ and sequenced by dideoxy Sanger method, performed onto 3130xl Genetic Analyzer (Applied Biosystem, Foster City CA, USA). Sequence reads obtained by Sanger were assembled with DNAStar software package (DNAStar Inc., Madison WI, USA). Representative H protein nucleotide (nt) sequences of canine distemper virus strains were retrieved from GenBank (Supplementary Table S2) and aligned using the Clustal Omega tool from the European Molecular Biology Laboratory (https://www.ebi.ac.uk/Tools/msa/clustalo/). Phylogenetic analysis was carried out by Mr Bayes program^48,49^ implemented within the software package Geneious (version 9.1.8, Biomatters Ltd., New Zealand). Bayesian inference was performed using four “chains” run over one million generations (with the first 2000 trees discarded as ‘burn-in) and supplying statistical support with subsampling over 200 replicates. jModelTest^50^ was used to identify the most appropriate model of evolution. The identified program settings for all partitions, under the Akaike Information Criteria, included six-character states (General Time Reversible model), a proportion of invariable sites, and a gamma distribution of rate variation across sites (GTR + I + G). H gene sequence of Phocine distemper virus was used as outgroup.

### Ethical Statement

All methods, including necropsy and sampling, were carried out in accordance with internal guidelines and regulations of IZSAM. All experimental protocols, including handling and processing of infectious tissues, were approved by the Ethical Committee of IZSAM.

## Supplementary information

Video S1

Supplementary Information
