# Supplementary material for: Diagnosis and characterization of canine distemper virus through sequencing by MinION nanopore technology
Source: Sci Rep. 2019 Feb 8;9:1714. doi: 10.1038/s41598-018-37497-4 (PMC6368598; doi:10.1038/s41598-018-37497-4)
Supplement: Supplementary file 2 — Supplementary Information [file 41598_2018_37497_MOESM2_ESM.docx]

**Supplementary Information**

**Diagnosis and characterization of canine distemper virus through sequencing by MinION nanopore technology**

Alessia Peserico^1^, Maurilia Marcacci^1^, Daniela Malatesta^1^, Marco Di Domenico^1^, Annamaria Pratelli^2^, Iolanda Mangone^1^, Nicola D’Alterio^1^, Federica Pizzurro^1^, Francesco Cirone^2^, Guendalina Zaccaria^1^, Cesare Cammà^1^, Alessio Lorusso^1*^

^1^National Reference Center for Whole Genome Sequencing of microbial pathogens: database and bioinformatic analysis, Istituto Zooprofilattico Sperimentale dell’Abruzzo e del Molise, Campo Boario, 64100 Teramo-Italy; ^2^Dipartimento di Medicina Veterinaria, strada provinciale per Casamassima Km 3, 70100 Valenzano, Bari-Italy

***Corresponding author:** Alessio Lorusso, [a.lorusso@izs.it](mailto:a.lorusso@izs.it), +390861332440

**Video S1, uploaded in the system**

**Supplementary Information legends**

**Video S1.** Severe neurological clinical signs of the infected dog including labored breathing, disorientation, lack of alertness, convulsive movements of the head and paws aimless wandering. Quarantine measures were applied. Clinical signs were taped by Dr. Dionisio Ianniruberto.

**Table S2.** Canine distemper virus strains employed for the phylogenetic analysis including lineage, GenBank accession number, strain name, host (organism from which virus was extracted), country and year of isolation.

| **Clade** | **Accession** | **Strain** | **Host** | **Country** | **Year** |
| --- | --- | --- | --- | --- | --- |
| Outgroup | AF479274 | Phocine | Baikal seal | DK | 2002 |
| America 1 | AB286953 | MD77 | dog | JP | 2006 |
| America 1 | AY548109 | 98-2655 | raccoon | US | 2004 |
| America 1 | AY548111 | 98-2666-2 | raccoon | US | 2004 |
| America 1 | AF259552 | Snyder Hill | dog | RU | 2000 |
| America 1 | DQ778941 | CDV3 | dog | CN | 2006 |
| America 1 | GQ332531 | 16 | dog | CN | 2008 |
| America 1 | HQ403645 | CDV-GZ1 | dog | CN | 2009 |
| America 1 | KY774572 | C53 | dog | CN | 2016 |
| America 1 | AM903376 | Indian | dog | IN | 2006 |
| America 1 | D00758 | Onderstepoort | vaccine | IR | 1991 |
| America 1 | Z35493 | Convac | vaccine | DK | 1994 |
| Rockborn | JX912968 | Bra-188/08 | dog | BR | 2008 |
| Rockborn | FJ461702 | VaccineD | vaccine | ZA | 2008 |
| Rockborn | GU810819 | Rockborn | dog | SE | 1959 |
| Rockborn | EF095750 | Hungary | vaccine | HU | 2006 |
| Rockborn | AY964114 | 25259 | dog | US | 2004 |
| Rockborn | AF178039 | lesser panda | lesser panda | CN | 1999 |
| Asia 1 | AB016776 | Tanu96 | raccoon dog | JP | 1998 |
| Asia 1 | AB286951 | MASS | dog | JP | 2006 |
| Asia 1 | GQ332532 | 17 | dog | CN | 2008 |
| Asia 1 | AB295487 | 81ND | dog | JP | 2007 |
| Asia 1 | DQ191175 | Canine/NTU 2004-1 | dog | TW | 2004 |
| Asia 1 | DQ887547 | Canine/NTU 2005-1 | dog | TW | 2005 |
| Asia 1 | EU296486 | TW/06-K127 | dog | TW | 2006 |
| Asia 1 | FJ705236 | TW7 | dog | TW | 2008 |
| Asia 1 | FJ705230 | TW1 | dog | TW | 2008 |
| Asia 1 | AY378091 | CD Taichung | dog | TW | 2003 |
| Asia 1 | EU325728 | JL (07)1 | raccoon dog | CN | 2007 |
| Asia 1 | JQ732173 | LDH (06) | fox | CN | 2006 |
| Asia 1 | FJ848530 | BJ080514 | dog | CN | 2008 |
| Asia 1 | EU325730 | LN (07)1 | raccoon dog | CN | 2007 |
| Asia 1 | FJ848535 | BJ080406 | dog | CN | 2008 |
| Asia 1 | AB212965 | S124C | dog | JP | 2005 |
| Asia 1 | D85755 | Yanaka | dog | JP | 1996 |
| Asia 1 | AB619774 | Yamaguchi/WT/100311 | tiger | JP | 2010 |
| Europe South America 1 | AF478548 | DK91 C | dog | DK | 2002 |
| Europe South America 1 | AF478550 | DK91 D | dog | DK | 2002 |
| Europe South America 1 | Z47761 | NA | dog | DK | 1995 |
| Europe South America 1 | AF478545 | B | dog | DK | 2002 |
| Europe South America 1 | Z77671 | 404 | dog | DE | 1994-95 |
| Europe South America 1 | Z77672 | 2544 | dog | DE | 1994-95 |
| Europe South America 1 | Z77673 | 4513 | dog | DE | 1994-95 |
| Europe South America 1 | DQ494317 | 324/03 | dog | IT | 2003 |
| Europe South America 1 | DQ494318 | 265/02-3 | dog | IT | 2003 |
| Europe South America 1 | DQ494319 | 111/03B | dog | IT | 2003 |
| Europe South America 1 | X85000 | 5804/Han90 | dog | UK | 1990 |
| Europe South America 1 | GU001863 | NA | Iberian lynx | SP | 2005 |
| Europe South America 1 | HM563057 | SMLM3 | wolf | PT | 1998 |
| Europe South America 1 | GQ214380 | 5417-02 | dog | AT | 2002 |
| Europe South America 1 | GQ214384 | 5825-02 | dog | AT | 2002 |
| Europe South America 1 | HM563058 | SMLM75 | wolf | PT | 2008 |
| Europe South America 1 | HM563059 | Lili | dog | PT | 2007 |
| Europe South America 1 | DQ889177 | H04Bp1F | dog | HU | 2006 |
| Europe South America 1 | JN153024 | 408/589/07 | red fox | DE | 2008 |
| Europe South America 1 | JN153025 | 458/07 | red fox | DE | 2008 |
| Europe South America 1 | HM120874 | SNP350/09/ITA | red fox | IT | 2009 |
| Europe South America 1 | JF810107 | W10/339 | red fox | SW | 2010 |
| Europe South America 1 | JQ966309 | MCDV216-L | marmot | SW | 2010 |
| Europe South America 1 | JF810109 | W10/2733 | lynx | SW | 2010 |
| Europe South America 1 | FJ416337 | BV2 | red fox | DE | 2008 |
| Europe South America 1 | FJ416338 | BV3 | badger | DE | 2008 |
| Europe South America 1 | FJ416339 | BV4 | red fox | DE | 2008 |
| Europe South America 1 | HM443726 | VIR3514 | red fox | IT | 2006 |
| Europe South America 1 | HM443708 | 09RS295 | stone martin | IT | 2009 |
| Europe South America 1 | HM443705 | 07RS1463 | red fox | IT | 2007 |
| Europe South America 1 | EU098103 | CDVBR2 | dog | BR | 2007 |
| Europe South America 1 | EU098104 | CDVBR3 | dog | BR | 2007 |
| Europe South America 1 | EU098105 | CDVBR4 | dog | BR | 2007 |
| Europe South America 1 | FJ392652 | Argentina 23 | dog | AR | 2003 |
| Europe South America 1 | EU098102 | CDVBR1 | dog | BR | 2007 |
| Europe South America 1 | JN215477 | Uy-141/09 | dog | UY | 2009 |
| Europe South America 1 | KM280689 | Uy251 | dog | UY | 2012 |
| Europe South America 1 | JN215476 | Uy-128/09 | dog | UY | 2009 |
| Europe South America 1 | JN215475 | Uy-111/08 | dog | UY | 2008 |
| Europe South America 1 | JN215473 | Uy-102/07 | dog | UY | 2007 |
| Europe South America 1 | JN215474 | Uy-109/08 | dog | UY | 2008 |
| America 2 | AF164967 | A75/17 | dog | SW | 1998 |
| America 2 | Z54166 | A92-6 | Black panther | NL | 1995 |
| America 2 | Z47764 | javelina | dog | DK | 1995 |
| America 2 | Z47762 | American dog | dog | DK | 1995 |
| America 2 | Z47765 | raccoon | raccoon | DK | 1995 |
| America 2 | Z54156 | 92-27/4 | Chinese leopard | DK | 1995 |
| European wildlife | Z47759 | Danish mink | mink | DK | 1995 |
| European wildlife | DQ889187 | H06Ny11 | dog | HU | 2006 |
| European wildlife | DQ889189 | H06Ny13 | dog | HU | 2006 |
| European wildlife | JN153019 | 124/07 | raccoon | DE | 2007 |
| European wildlife | JN153023 | 236/07 | raccoon | DE | 2007 |
| European wildlife | JN153021 | 140/07 | raccoon | DE | 2007 |
| European wildlife | KX545421 | CDV599/2016 | red fox | IT | 2016 |
| South America 2 | FJ011005 | Bruno107Arg2005 | dog | AR | 2005 |
| South America 2 | FJ392651 | Argentina 24 | dog | AR | 2005 |
| South America 2 | KC257464 | Argentina 26 | dog | AR | 2010 |
| U.S. wildlife | AB301066 | Th270L | dog | TH | 2007 |
| U.S. wildlife |  | Cuba |  |  |  |
| U.S. wildlife | AY964110 | 19876 | dog | US | 2005 |
| Colombian | KF835425 | 44-CO/12 | dog | CO | 2012 |
| Colombian | KF835414 | 18-CO/12 | dog | CO | 2012 |
| Colombian | KF835421 | 40-CO/12 | dog | CO | 2012 |
| Colombian | KF835418 | 30-CO/12 | dog | CO | 2012 |
| Colombian | KF835424 | 42-CO/12 | dog | CO | 2012 |
| Colombian | KF835412 | 13-CO/12 | dog | CO | 2012 |
| Colombian | KF835423 | 41-CO/12 | dog | CO | 2012 |
| Colombian | KF835420 | 34-CO/12 | dog | CO | 2012 |
| Colombian | KF835419 | 33-CO/12 | dog | CO | 2012 |
| Colombian | KF835417 | 29-CO/1 | dog | CO | 2012 |
| Colombian | KF835416 | 26-CO/12 | dog | CO | 2012 |
| Colombian | KF835415 | 19-CO/12 | dog | CO | 2012 |
| Colombian | KF835411 | 2-CO/11 | dog | CO | 2011 |
| Colombian | KF835413 | 14-CO/12 | dog | CO | 2012 |
| Colombian | KF835422 | 39-CO/12 | dog | CO | 2012 |
| Colombian | X84999 | 1493/Han89 | ferret | RU | 1989 |
| Asia 2 | JQ732170 | ZH (05) | raccoon | CN | 2005 |
| Asia 2 | AB025270 | 98-002 | dog | JP | 1998 |
| Asia 2 | AB295480 | 03Cbr | dog | JP | 2007 |
| Asia 2 | AB295486 | 66L | dog | JP | 2007 |
| Asia 2 | AB212729 | 007LmT | dog | JP | 2005 |
| Asia 2 | AB250668 | 007Lm-20p | dog | JP | 2006 |
| Asia 2 | AB252717 | 011C | dog | JP | 2006 |
| Asia 2 | AB252718 | 009L | dog | JP | 2006 |
| Asia 2 | AY297453 | 5B | dog | JP | 2003 |
| Asia 2 | AY297454 | 5VD | dog | JP | 2003 |
| Asia 2 | EU252148 | Seoul | dog | KR | 2007 |
| Asia 2 | AB040767 | HM-3 | dog | JP | 2000 |
| Asia 2 | EU716074 | 98Marten | marten | KR | 1998 |
| Asia 2 | EU716075 | 07D111 | dog | KR | 2007 |
| Asia 2 | EU716073 | 97Jindo | dog | KR | 1997 |
| Asia 2 | FJ868161 | C2 | dog | KR | 2009 |
| Asia 2 | FJ868169 | R4 | raccoon | KR | 2009 |
| Asia 2 | EU252149 | Chunchon | dog | KR | 2007 |
| Asia 2 | AB040768 | HM-6 | dog | JP | 2000 |
| Africa | FJ461693 | 4L70214 | dog | ZA | 2007 |
| Africa | FJ461695 | 13sp | dog | ZA | 2007 |
| Africa | FJ461721 | 12sp | dog | ZA | 2007 |
| Africa | FJ461714 | 4L7039 | dog | ZA | 2007 |
| Africa | FJ461715 | 4sp | dog | ZA | 2007 |
| Africa | FJ461718 | 5sp | dog | ZA | 2007 |
| Africa | FJ461723 | 23sp | dog | ZA | 2007 |
| Africa | FJ461722 | 23L | dog | ZA | 2007 |
| Africa | FJ461716 | 16L | dog | ZA | 2007 |
| Africa | FJ461720 | 2L | dog | ZA | 2007 |
| Africa | FJ461694 | 21L | dog | ZA | 2007 |
| Africa | FJ461711 | 7L | dog | ZA | 2007 |
| Africa | FJ461696 | 1sp | dog | ZA | 2007 |
| Arctic-like | KF914669 | CDV2784/2013 | dog | IT | 2013 |
| Arctic-like | KC966928 | Wa-CDV2013 | wolf | IT | 2013 |
| Arctic-like | KC966929 | Wb-CDV2013 | wolf | IT | 2013 |
| Arctic-like | KX024708 | CDV11956/2015 | badger | IT | 2015 |
| Arctic-like | KX024709 | CDV12254/2015 | badger | IT | 2015 |
| Arctic-like | HM443706 | 08RS2382 | dog | IT | 2010 |
| Arctic-like | DQ889183 | H05Bp7F | dog | HU | 2006 |
| Arctic-like | DQ889184 | H06Bp8F | dog | HU | 2006 |
| Arctic-like | DQ889185 | H06Bp9S | dog | HU | 2006 |
| Arctic-like | HM443722 | 6897 | dog | IT | 2002 |
| Arctic-like | HM443724 | 9513 | dog | IT | 2002 |
| Arctic-like | DQ226087 | 179/94 | dog | HU | 2006 |
| Arctic-like | HM443715 | 323 | dog | IT | 2004 |
| Arctic-like | HM443721 | 6291 | dog | IT | 2002 |
| Arctic-like | HM443712 | 2283 | dog | IT | 2005 |
| Arctic-like | DQ226088 | 48/05 | dog | HU | 2006 |
| Arctic-like | AY964112 | 21261 | dog | US | 2005 |
| Arctic-like | AY964108 | 18133 | dog | US | 2005 |
| Arctic-like | HM443714 | 3204 | dog | IT | 2000 |
| Arctic-like | HM443716 | 4454 | dog | IT | 2000 |
| Arctic-like | HM443711 | 193 | dog | IT | 2001 |
| Arctic-like | HM443719 | 5735 | dog | IT | 2000 |
| Arctic-like | HM443713 | 2894 | dog | IT | 2000 |
| Arctic-like | AF172411 | liud | dog | CN | 1999 |
| Arctic-like | EF445052 | HL | red fox | CN | 2007 |
| Arctic-like | HM443710 | 1900 | dog | IT | 2000 |
| Arctic-like | Z47760 | Greenlandic dog | dog | DK | 1995 |
| Arctic-like | X84998 | PDV-2 | Baikal seal | RU | 1995 |
